# Healthcare facility factors associated with health-seeking behavior among secondary school students in the Dodoma region: an analytical cross-sectional study

**DOI:** 10.3389/fpubh.2025.1457318

**Published:** 2025-03-27

**Authors:** Karim Faragha Athumani, Joanes Faustine Mboineki

**Affiliations:** School of Nursing and Public Health, The University of Dodoma, Dodoma, Tanzania

**Keywords:** health-seeking behavior, adolescents, factors, health facilities, secondary school students

## Abstract

**Background:**

Health-seeking behavior (HSB) is an important aspect of population health and is closely linked to a nation’s economic development. Its importance is reflected in the Sustainable Development Goal 3. Although previous studies in Tanzania have examined health-seeking behavior, the majority of them have focused on the behavior of seeking medical attention after becoming sick. The present study assessed healthcare facility factors associated with health-seeking behavior among secondary school students in the Dodoma region.

**Methodology:**

This was a cross-sectional analytical study that included 311 adolescent secondary school students. The data were collected through a validated questionnaire and were analyzed using descriptive statistics and chi-squared test values (χ^2^).

**Results:**

Among the 311 study respondents, 62 (19.9%) were found to exhibit health-seeking behavior. Healthcare facility factors associated with adolescents’ health-seeking behavior included healthcare service costs (χ^2^ = 12.415, *p* = 0.015) and long queues (χ^2^ = 10.077, *p =* 0.039).

**Conclusion:**

The health-seeking behavior among adolescents was not satisfactory and is influenced by factors such as service costs, long queues, and their socio-demographic characteristics (age, sex, and education). Improving adolescents’ health-seeking behavior requires reducing hospital waiting times and queues. To address cost barriers to health-seeking behavior, initiatives should be implemented to ensure that each secondary school student has health insurance. Interventions need to consider adolescents’ age, sex, and education to improve their health-seeking behavior. For instance, priorities should be directed toward very young adolescents and those with lower levels of education.

## Introduction

Health-seeking behavior (HSB) is an important aspect of population health as it is closely linked to a nation’s economic development (1). Its importance is reflected in Sustainable Development Goal 3, which ensures healthy lives and promotes well-being for everyone at all ages (2). Moreover, HSB underpins the implementation of Universal Health Coverage programs (3). Poor health-seeking behavior is associated with increased mortality, even for easily manageable conditions (4), increased morbidity, higher community transmission risks, worse health outcomes, and poor health statistics (5).

Although HSB is important for individuals of all age groups, it is particularly important for the young, as this is a unique stage of human development and serves as a vital period for establishing healthy habits (6). Furthermore, the existing literature reports on health-seeking behavior in the general population and university students, with limited documentation on secondary school students. Therefore, the current study focused on secondary school students enrolled in the second stage of formal education, typically between the ages of 12 and 18 years (7). Secondary school students constitute 25% of the population (8), and they fall within the World Health Organization category of adolescents, which is the phase of life between childhood and adulthood, from ages 10 to 19 years (6). This age group is at risk of HIV, other sexually transmitted diseases (STDs), and unintended pregnancy (9). This is an age group with a high prevalence of mental health problems, such as depression, anxiety disorders, emotional and behavioral difficulties, post-traumatic stress, and suicidal behavior (10). In addition, they encounter non-communicable diseases (NCDs) due to harmful behavioral practices such as smoking, unhealthy diet patterns, and low levels of physical activity (11).

Health-seeking behavior remains unsatisfactory in many regions. For example, in India, only 27.9% of students with health problems were reported to consult doctors (12), while in South Africa, approximately 27% were reported to seek healthcare (13). It was reported that 58% of students were reluctant to seek help for mental health (14), while 76.6% never visited health facilities for sexual and reproductive health issues, and 56.0% were not willing to visit a health facility if they ever had any sexual or reproductive health issues (15).

Healthcare-seeking behavior refers to any activity undertaken by individuals who perceive they are ill or have a health problem in order to obtain an appropriate remedy (16). Moreover, health-seeking behavior encompasses activities undertaken to maintain good health, prevent ill health, and deal with any deviation from a favorable state of health (1). Based on this study, the operational definition of health-seeking behavior referred to an individual seeking medical attention even without signs and symptoms. It involved seeking preventive services, such as vaccinations or check-ups from healthcare facilities.

Several factors hindering health-seeking behavior among adolescents have been reported, including the negative attitude of nurses, negative attitude of the community, peer influence, shortage of medicine, lack of services over the weekend, long queues, waiting times, distance, and lack of knowledge (17). In addition, the high treatment cost has been identified as a factor hindering adolescents’ health-seeking behavior (18). Despite the documented factors for health-seeking behavior, the majority of them are individual factors. It is recommended that scholars investigate how much the healthcare system influences health-seeking behavior (4). Therefore, this study focused on healthcare facility factors related to HSB. The Andersen healthcare utilization model was used to formulate a conceptual framework showing relationships between variables. The model aims to demonstrate how the constructs—(i) predisposing factors (age, sex, religion, and education), (ii) enabling factors (healthcare costs, distance to healthcare facilities, healthcare providers’ attitude, and long queues), and (iii) healthcare utilization, which is health-seeking behavior—interact.

In Tanzania, 68% of the population does not seek healthcare (19). Traditional values, norms, accessibility to healthcare services, poor awareness due to inadequate sources of information, and lack of resources all affect adolescents’ health-seeking behavior (20). Even though previous studies have reported on factors associated with health-seeking behavior, very few have focused on healthcare facility factors leading to health-seeking behavior. Meanwhile, most of the previous studies have focused on the behavior of seeking medical attention after becoming sick and have concentrated on the general population rather than secondary school students. The present study aimed to assess healthcare facility factors associated with health-seeking behavior among secondary school students in the Dodoma region. The study had the following three specific objectives: (1) to determine the prevalence of health-seeking behavior among adolescents in the Dodoma region, (2) to assess healthcare facility factors associated with health-seeking behaviors of adolescents in the Dodoma region, and (3) to assess social demographic factors associated with health-seeking behavior among adolescents in the Dodoma region. Furthermore, the study included three research questions: (i) What is the prevalence of health-seeking behavior among adolescents in the Dodoma region? (ii) What healthcare facility factors are associated with health-seeking behavior among adolescents in the Dodoma region? (iii) What socio-demographic factors are associated with health-seeking behavior among adolescents in the Dodoma region?

## Methods

### Study design

This was a cross-sectional analytical study designed to assess the current health-seeking behavior and factors associated with that behavior. This design is the most relevant for estimating the prevalence of HSB and assessing the association between different parameters (21).

### Study setting

The study was conducted in two secondary schools located in the Dodoma municipal district. Secondary schools were selected because the majority of the adolescents can be found. Furthermore, Dodoma is reported as a leading region in Tanzania with a higher rate of teenage pregnancies, ranging from 34 to 45% (22). Dodoma has also been considered a region where adolescents experience malnutrition (23), alcohol abuse, and psychological problems, triggering the need for health-seeking behavior (24).

### Study population

The study population included all secondary school adolescent male and female students aged 10–19 years in the Dodoma municipal district.

### Inclusion criteria

Adolescent students aged 10–19 years from the two selected public secondary schools who were mentally stable and able to read and write were selected to participate in the study. Students were excluded if they were unwilling to participate in the study.

### Sample size calculation and sampling technique

The sample size was computed using G*Power version 3.1.9.4, with the test family set to *X*^2^ and the statistical test selected as the goodness-of-fit test. The effect size was 0.2526, the power was 95%, and the α err prob. was 0.05. The computed sample size for the study was 239 adolescents. Simple random sampling (25) was used to select the district, schools, and participants. For instance, using a lottery method, one district (Dodoma municipal) was selected out of seven districts and two public secondary schools were chosen out of 36 schools. A proportionate sampling technique (26) was used to determine the number of participants to be recruited from each school. Simple random sampling using the lottery method was also applied to select participants from the two public secondary schools.

### Data collection procedures and data collection tools

The study was conducted from 16 June 2022 to 30 July 2022 using a validated questionnaire. Self-administered and interviewer-administered questionnaires were used as data collection approaches. Interviewer-administered questionnaires were specifically employed for the participants who required additional assistance. A thorough review was performed to confirm that all questions were completed.

The questionnaire used to assess healthcare facility factors influencing health-seeking behavior was adapted from a previous study by Rango (27). The tool was modified to reflect the local context of Tanzania. A psychometric validation of the tool was carried out through a validity and reliability process. The validity was assessed through face validity and content validity, where a few secondary school students were asked if they encountered difficulties filling out the questionnaire and experts were asked to rate the quality of the tool and provide comments, respectively. Based on the face validity results, the language of a few items was recommended to be simplified. Meanwhile, the content validity assessment revealed that the experts expressed high satisfaction with the tool’s content and adequacy, although they suggested revising the sequences of the items. The comments and suggestions obtained from the face and content validity assessments were addressed, and the tool was revised. Regarding reliability, internal consistency reliability was performed, where the tool was pretested among 93 secondary school students.

The pretest was conducted by the principal investigator at a single school in Dodoma, which was not included in the actual study. The students recruited for the pretest were aged 10–19 years. The pretest data were entered into the Statistical Package for the Social Sciences (SPSS), and the reliability was computed. The Cronbach’s alpha value was above 0.7, indicating that the tool was appropriate. The final draft of the questionnaire was translated into Swahili, the native language, so that the participants could comprehend the questions. The questionnaire consisted of two sections: section A (sociodemographic characteristics of the respondents) and section B (factors influencing health-seeking behavior). Some questions in section B were based on a 4-point Likert scale (Strongly agree, agree, strongly disagree, and disagree).

### Variable definitions and measurements

Since the study utilized the Andersen healthcare utilization model, three constructs guided the study in establishing relationships between variables. One of the constructs is predisposing factors, which are defined as demographic characteristics and social structural variables pertaining to health services (28). This construct includes variables such as age, sex, religion, and education. Age was measured as a continuous variable, while sex and religion were measured as nominal variables. The second construct is enabling factors, which are defined as resources available, whether individually or in a community, for healthcare utilization. The enabling factors include healthcare costs, distance to healthcare facilities, healthcare providers’ attitude, and long queues, all of which were assessed using a 4-point Likert scale. Meanwhile, healthcare utilization was assessed by asking the respondents if they had health-seeking behavior.

### Data analysis plan

The data collected from the field were analyzed using SPSS version 26. Descriptive statistics were performed to identify the frequencies and percentages. The association of the variables was analyzed through cross-tabulation, yielding a chi-square test value (χ^2^) and a probability value (*P*). The significance level was set at 0.05.

### Ethical consideration

An ethical clearance letter was obtained from the University of Dodoma Institutional Research Review Committee (IRREC). The School of Nursing and Public Health of the University of Dodoma provided the introduction letter. A permission letter to conduct the study at the selected schools was provided by the Dodoma City Executive Director. Each participant completed a consent form before data collection. For the participants under 18 years, their legal guardian completed the consent form, and informed consent was obtained from the legal guardian. The participants’ confidentiality was maintained by avoiding the use of their names. The participants were free to join or withdraw from the study whenever they felt like doing so. We declare that all the described methods were performed according to the relevant guidelines.

## Results

### Social demographic data characteristics of the sample

The average age of the respondents in the study was 16.2 years, with a standard deviation of 1.3. The minimum age was 14 years, and the maximum age was 19 years. The number of male individuals who responded to the study was 167 (53.7%), while the number of female individuals who responded was 144 (46.3%). In terms of religion distribution, 247 (79.4%) were Christians, 52 (16.7%) were Muslims, and 12 (3.9%) identified as others. Refer to Table 1.

**Table 1:** Sociodemographic characteristics of the adolescents.

Variables	Frequency (n)	Percentage (%)
**Age**		
The mean age of the participant was 16.2, SD of 1.3, 14–19 years		
**Sex**		
Male subjects	167	53.7
Female subjects	144	46.3
**Religion**		
Christian	247	79.4
Muslim	52	16.7
Other	12	3.9
**Level of education**		
Form four	93	29.9
Form three	94	30.2
Form two	68	21.9
Form one	56	18
Total	311	100

### Prevalence of health-seeking behavior

Among the 311 study respondents, 62 (19.9%) were found to have health-seeking behavior, while 249 (80.1%) had no health-seeking behavior.

### Healthcare facility factors associated with adolescents’ health-seeking behavior

Participants were asked whether healthcare service costs influence health-seeking behavior. Among them, 176 (56.6%) strongly agreed, 62 (19.9%) agreed, 37 (11.9%) strongly disagreed, and 30 (9.6%) disagreed. After cross-tabulation, the result showed χ^2^ = 12.415 and *p* = 0.015. The participants responded that the distance to healthcare facilities can influence their health-seeking behavior. It was found that 106 (34.1%) participants strongly agreed, 94 (30.2%) agreed, 55 (17.7%) disagreed, and 50 (16.1%) strongly disagreed. There was no significant association between distance and health-seeking behavior, with χ^2^ = 5.45 and *p =* 0.244. The participants were asked if healthcare providers possess a negative attitude during the delivery of care, 99 (31.8%) participants agreed, 81 (26.0%) strongly agreed, 76 (24.4%) disagreed, and 55 (17.7%) strongly disagreed. After cross-tabulation, there was no significant association between the negative attitude of healthcare providers and health-seeking behavior (χ^2^ = 3.335, *p =* 0.343). Furthermore, the participants were asked whether long queues are also a factor influencing adolescents’ health-seeking behavior. Of the respondents, 136 (43.7%) disagreed, 83 (26.7%) strongly disagreed, 56 (18.0%) agreed, and 30 (9.6%) strongly agreed. The association between long queues and health-seeking behavior among the adolescents was significantly different (χ^2^ = 10.077, *p =* 0.039). Refer to Table 2.

**Table 2:** Healthcare facility factors associated with adolescents’ health-seeking behavior.

Variables	Frequency (n)	Percentage (%)	*χ* ^2^	*p*-value
**Healthcare costs**				
Strongly agree	176	56.6	12.415	0.015
Agree	62	19.9		
Disagree	30	9.6		
Strongly disagree	37	11.9		
**Distance to healthcare facilities**				
Strongly agree	106	34.1	5.45	0.244
Agree	94	30.2		
Disagree	55	17.7		
Strongly disagree	50	16.1		
**Healthcare providers’ negative attitude**				
Strongly agree	81	26.0	3.335	0.343
Agree	99	31.8		
Strongly disagree	55	17.7		
Disagree	76	24.4		
**Long queues**				
Strongly agree	30	9.6	10.077	0.039
Agree	56	18.0		
Strongly disagree	83	26.7		
Disagree	136	43.7		

### Association between adolescents’ social demographic data and their health-seeking behavior

It was found that the age of adolescents was significantly associated with their health-seeking behavior (*p* < 0.001), and the adolescents’ sex was statistically significant (*p =* 0.006). Furthermore, their education level was also statistically significant regarding health-seeking behavior (*p =* 0.032).

## Discussion

### Prevalence of health-seeking behavior among secondary school students

The present study found that only 19.9% of the participants exhibited health-seeking behavior. This indicates that a small percentage of secondary school students have the habit of seeking medical attention. A similar small percentage was reported in a previous study, where 27.9% of secondary school students consulted doctors when they felt sick (12). This is consistent with a previous mixed-method study, which reported that more than 30% of adolescents did not seek medical attention even when they knew it was needed (29). In addition, the findings of a previous descriptive cross-sectional study in Nigeria support the current study, showing that 63.5% of adolescent schoolgirls had never visited a health facility for STIs, while 36.5% had sought treatment (30).

### Healthcare facility factors associated with adolescents’ health-seeking behavior

The study found that healthcare service costs and long queues are healthcare facility factors significantly associated with secondary school students’ health-seeking behavior. Regarding costs, the findings are similar to those of a previous study, which indicated that the cost of services at healthcare facilities is a major obstacle to health-seeking behavior (31). Most of the secondary school students come from low-income families, making them unable to seek medical services that require payments, even if they have the intention of doing so. Poverty is evident for most parents and was one of the reasons the Tanzania government decided to abolish fees and other contributions for secondary schools, aiming to increase access to secondary education for the youth (32). In addition to poverty, the students might not have health insurance to cover diagnostic procedures and treatment.

The significant findings regarding the impact of long queues on health-seeking behavior among secondary school students are consistent with those of a previous study by Ripfumelo (17). Long queues increase waiting times, compromise the quality of patient care, and heighten dissatisfaction with healthcare services (33). Since the average waiting time to receive services in Tanzania is long, ranging from 3 to 4 h (34, 35), it may discourage secondary school students from seeking medical services due to their busy academic schedules.

### Association between adolescent secondary school students’ social demographic data and their health-seeking behavior

Adolescents’ age, sex, and education level were statistically significant factors in health-seeking behavior. As adolescents grow older, they are more likely to be exposed to various sources of information, such as media and peers. With age, adolescent secondary school students can better comprehend the information, assess their health-related reality, and engage in self-care. Secondary schools in Tanzania have curricula that incorporate adolescent sexual health education in Form Two and Form Three. Therefore, students in Form Two and above are more likely to exhibit health-seeking behavior. The previous findings align with the present study, indicating that age, sex, and education levels influence health-seeking behavior (36).

### Study limitations

The current study adopted an analytical cross-sectional design, which is well-suited for testing assumptions about relationships. However, a key limitation of this design is its inability to separate a presumed cause from its possible effect. Two concepts may correlate significantly, but this does not mean that one causes the other. In addition, the absence of a neutral response in the Likert scale items may have reduced the reliability and validity of the findings.

## Conclusion

The health-seeking behavior among adolescents was not satisfactory and affected by service costs, long queues, and their sociodemographic characteristics (age, sex, and education). Improving adolescents’ health-seeking behavior requires reducing hospital waiting times and queues. To address cost barriers to health-seeking behavior, initiatives should be implemented to ensure that each secondary school student has health insurance. Interventions need to consider adolescents’ age, sex, and education to improve their health-seeking behavior. For instance, priorities should be directed toward very young adolescents and those with lower levels of education.

## Data Availability

The original contributions presented in the study are included in the article/supplementary material, further inquiries can be directed to the corresponding author.
